# Estimation of Parameters Obtained by Electrochemical Impedance Spectroscopy on Systems Containing High Capacities

**DOI:** 10.3390/s90907365

**Published:** 2009-09-11

**Authors:** Zoran Stević, Mirjana Rajčić Vujasinović, Milan Radunović

**Affiliations:** 1 University of Belgrade, Technical Faculty in Bor, VJ 12, 19210 Bor, Serbia; E-Mail: mrajcic@tf.bor.ac.rs (M.R.V.); 2 Faculty FUTURA, University Singidunum, Bulevar Mihajla Pupina 12a, 11070 New Belgrade, Serbia; E-Mail: milan.radunovic@encoen.rs (M.R.)

**Keywords:** electrochemical measurements, measurement system, electrochemical impedance spectroscopy, supercapacitors

## Abstract

Electrochemical systems with high capacities demand devices for electrochemical impedance spectroscopy (EIS) with ultra-low frequencies (in order of mHz), that are almost impossible to accomplish with analogue techniques, but this becomes possible by using a computer technique and accompanying digital equipment. Recently, an original software and hardware for electrochemical measurements, intended for electrochemical systems exhibiting high capacities, such as supercapacitors, has been developed. One of the included methods is EIS. In this paper, the method of calculation of circuit parameters from an EIS curve is described. The results of testing on a physical model of an electrochemical system, constructed of known elements (including a 1.6 F capacitor) in a defined arrangement, proved the validity of the system and the method.

## 1. Introduction

Every system may be regarded in a frequency domain displaying frequency logarithm on the X–axis and logarithm of module and/or phase angle of transfer function on the Y–axis (Bode plot) [1–3]. For the electrochemical system, the transfer function is, in fact, the impedance of an equivalent electrical circuit i.e., its complex form. That is why this method is named electrochemical impedance method (EIS) and it is widely used for characterization of electrochemical systems [4–14]. Applying alternate voltage, U(S), of different frequencies and constant amplitude to an electrical circuit, responding current, I(S), will appear [2,3]. Amplitude and phase angle of this current will depend on voltage and impedance, Z(S), of the circuit at that frequency:

$$I(S)=\frac{U(S)}{Z(S)}$$

where S = σ + jω is Laplace complex variable. For sinusoidal excitation the real part σ is equal to zero, so the S becomes S = jω, where frequency, ω, is given in s^−1^.

Conventional impedance spectra are actually snapshots of transfer functions taken at certain well-defined states of the system (usually stationary, constant potential states). However, for a fuller description of electrochemical systems the evolution of impedance spectra should be investigated during the evolution of the system in both potential and time. With the progress of digital techniques this is becoming increasingly possible [15–17].

The system used for electrochemical measurements consisted of hardware (PC, AD-DA converter NI–621 produced by National Instruments and an analog interface developed at the Technical Faculty in Bor) and software for excitation and measurement (LABVIEW platform and originally developed application software) [18,19]. With the goal of achieving a full mathematical analysis of the measured data directly in the LabVIEW application, it was necessary to develop our own mathematical model which is implemented in the measurement software and described in this work. This was not possible with some commercially available fitting software packages (EqCwin, Z-view) [20].

The possibilities of the software described here are compared with the Thales software of the Zahner EIS firm [21,22]. Our model and software are more adequate to the real system because the model better describes real electrochemical systems taking into account the complexity of the processes. The mathematical model developed herein is adapted to the investigated class of electrochemical systems and it is strongly connected with the physical parameters of the system. That approach enabled us to obtain analytical values of mutual relationships between the physical parameters from the system response and, in that way, to make system optimization following some given criteria. This is a significant advantage in compare to the commercial software, where the model is not “visible”.

## 2. Theoretical Part

By recording amplitude and phase angle of the response current for every frequency value (excitation voltage known), one can obtain the module and the phase angle of impedance for that frequency; this is presented as one point on the Bode plot which gives the dependence of impedance module, Z, on frequency, f, in logarithmic scale. Logarithm is used in a goal to obtain linear dependences instead of exponential ones. At frequencies obtained by extrapolation of straight segments, some deviation from straight line appears, and the line slope changes gradually. From the heights of the horizontal regions and corner frequencies, one can calculate all the parameters of the circuit of which the Bode plot is recorded, i.e. to estimate the equivalent circuit parameters [23–25].

**Figure 1.**
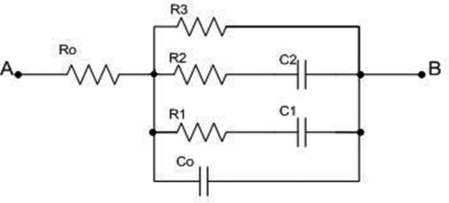
Considered equivalent electrical circuit.

After years of investigating of electrochemical behavior of different electrode materials, different equivalent electrical circuits that exhibit the same response on excitations as considered electrochemical systems have been found [23–26]. One of the most common was the circuit presented in Figure 1.

R_0_ corresponds to the resistance of electrolyte and electrode material, and its value is on an order of magnitude of milliohms (mΩ) or Ohms (Ω). Capacity C_0_ corresponds to double layer formed on the electrolyte side. Resistances R_1_ and R_2_ (order of magnitude ohm to tens Ohms) are related to slow processes of adsorption and diffusion, as well as the capacitances C_1_ and C_2_. As a matter of fact, the branch R_1_C_1_ exhibits and describes the inconstancy of parameters in R_2_C_2_ branch. R_3_ is resistance of self-discharging, meaning that it is reciprocal to leakage current. Its value is on the order of hundreds of Ohms to tens of kiloohms.

**Figure 2.**
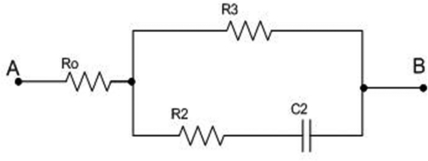
Equivalent circuit for the second frequency domain (on the order of mHz).

For the adopted equivalent circuit (Figure 1) in a general case the impedance equation is complex and not clear enough. So, here a step by step method is applied, one frequency domain after other, knowing the nature of the process, i.e. orders of magnitude of the circuit parameters. For very low frequencies (on the order of μHz) all capacitors do not conduct electricity, so the impedance of the circuit remains the serial connection of R_0_ and R_3_:

$$Z_{1}=R_{0}+R_{3}$$

where Z_1_ is correlated to the first (the highest) horizontal plateau in Figure 5. At frequencies on the order of mHz capacitor C_2_ conducts, while C_1_ and C_0_ still are infinite resistances; so, the equivalent circuit has the shape presented in Figure 2.

The impedance of the circuit presented in Figure 2 is:

$$Z=\frac{S[(R_{1}+R_{3})\cdot R_{0}C_{2}+R_{2}R_{3}C_{2}]+R_{0}+R_{3}}{{\text{SC}}_{2}(R_{2}+R_{3})\;+\;1}$$

From the conditions for the impedance zero and pole, the corner frequencies may be obtained as:

$$f_{1}=\frac{1}{2\pi \cdot (R_{2}+R_{3})\cdot C_{2}}$$

$$f_{2}=\frac{R_{0}+R_{3}}{2\pi \cdot [(R_{2}+R_{3})\cdot R_{0}C_{2}+R_{2}R_{3}C_{2}]}$$

At some higher frequencies (in order of dozens mHz) C_2_ becomes short circuit, while C_0_ and C_1_ are still in break, so the height of this horizontal region is:

$$Z_{2}=R_{0}+R_{23}\;\;\;\text{where}\;R_{23}=\frac{R_{2}R_{3}}{R_{2}+R_{3}}$$

**Figure 3.**
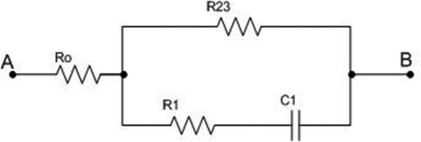
Equivalent circuit for fourth frequency domain (on the order of hundreds of mHz).

At frequencies on the order of hundreds of mHz, C_1_ starts conducting, C_0_ is still in break, and C_2_ is a short circuit; the equivalent circuit then has the shape given in Figure 3.

The impedance of the circuit is then:

$$Z=\frac{S[(R_{1}+R_{23})\cdot R_{0}C_{1}+R_{23}R_{1}C_{1}]+R_{0}+R_{23}}{{\text{SC}}_{1}(R_{1}+R_{23})+1}$$

From the previous equation, corner frequencies may be obtained as:

$$f_{3}=\frac{1}{2\pi \cdot (R_{1}+R_{23})\cdot C_{1}}$$

and

$$f_{4}=\frac{R_{0}+R_{23}}{2\pi \cdot [(R_{1}+R_{23})\cdot R_{0}C_{1}+R_{23}R_{1}C_{1}]}$$

Next horizontal region is obtained at frequencies higher then 1 Hz, when capacitor C_1_ becomes a short circuit, as well as C_2_, while C_0_ still does not conduct; so it can be written:

$$Z_{3}=R_{0}+R_{123}$$

where R_123_ is a parallel connection of R_1_, R_2_ and R_3_.

**Figure 4.**
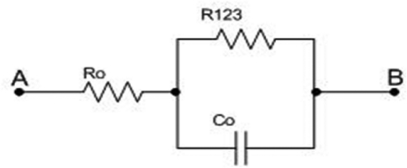
Equivalent circuit for sixth frequency domain (on the order of kHz).

At relatively high frequencies (on the order of kHz) C_0_ starts leading, while C_1_ and C_2_ are short circuits, so the equivalent circuit becomes as in Figure 4.

The impedance of such circuit is:

$$Z=\frac{{\text{SR}}_{0}R_{123}C_{0}+R_{0}+R_{123}}{{\text{SR}}_{123}C_{0}+1}$$

and corner frequencies are:

$$f_{5}=\frac{1}{2\pi R_{123}C_{0}}$$

$$f_{6}=\frac{R_{0}+R_{123}}{2\pi R_{0}R_{123}C_{0}}$$

At the end, the lowest horizontal part of Bode plot is obtained at highest frequencies (on the order of tens of kHz) when C_0_ is in short circuit, too, so:

$$Z_{4}=R_{0}$$

**Figure 5.**
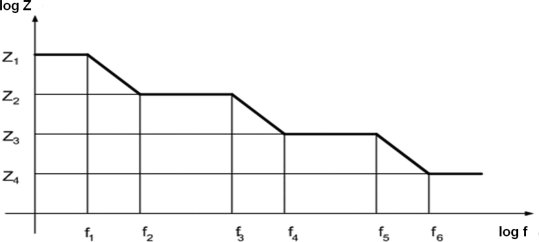
Theoretical Bode plot for adopted equivalent circuit.

The theoretical Bode plot for the whole equivalent circuit given in Figure 1 is presented in Figure 5.

## 3. Experimental

Testing of the system and developed method was done on a physical model of the electrochemical system, constructed of known elements in a defined arrangement as in Figure 1.

**Figure 6.**
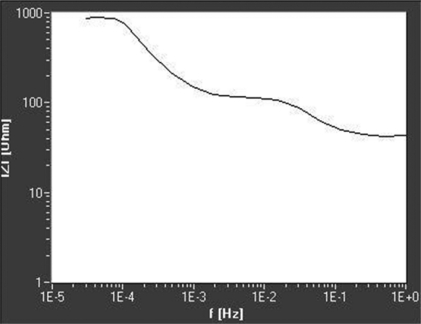
Experimentaly obtained Bode plot for the physical model (R_3_ = 1 kΩ).

**Figure 7.**
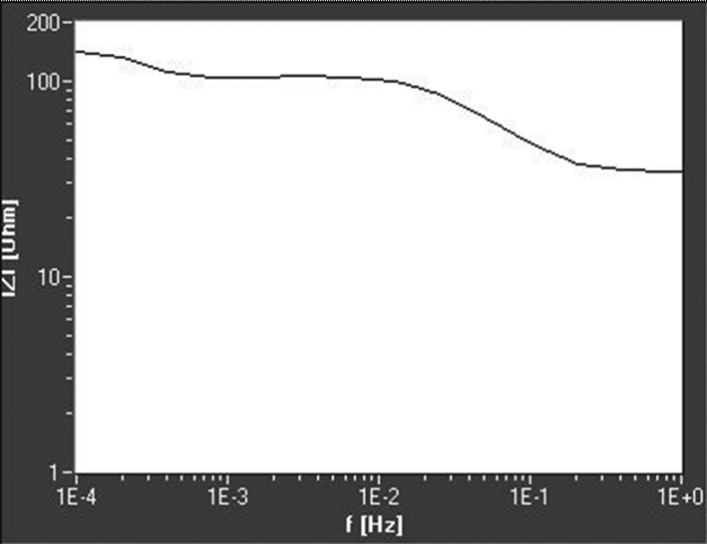
Experimentaly obtained Bode plot for the physical model (R_3_ = 150 Ω).

The elements that the physical model was made of were: R_0_ = 3 Ω; R_1_ = 39 Ω, R_2_ =90 Ω; C_0_ = 0,12 μF; C_1_ = 30 mF; C_2_ = 1,6 F and R_3_ = 1 kΩ (alternatively R_3_ = 150 Ω). Experiments were performed using the following parameters: DC level 10 mV, AC amplitude 5 mV, frequency range 30 μHz up to 1 Hz. The obtained curves are presented in Figures 6 and 7.

From the experimentally obtained Bode curve, all parameters of the system have been determined by following the next steps:

From the plateau 4, R_0_ is obtained immediately from R_0_ = Z_4_;

Horizontal region 1 is equal to Z_1_, and then R_3_ can be calculated from:

$$R_{3}=Z_{1}-R_{0}$$

Plateau 2 gives Z_2_, and then applying:

$$R_{23}=Z_{2}-R_{0}\;\text{and}\;R_{2}=\frac{R_{23}R_{3}}{R_{3}+R_{23}}$$

From horizontal part 3, we get Z_3_ and calculate R_123_ = Z_3_ – R_0_. Then R_1_ can be estimated from:

$$R_{1}=\frac{R_{123}R_{23}}{R_{23}+R_{123}}$$

From the corner frequency f_1_, capacitance C_2_ is calculated from:

$$C_{2}=\frac{1}{2\pi f_{1}\cdot (R_{2}+R_{3})}$$

From the corner frequency f_3_, C_1_ can be calculated as:

$$C_{1}=\frac{1}{2\pi f_{3}\cdot (R_{1}+R_{23})};$$

Finally, from the corner frequency f_5_, C_0_ is estimated as:

$$C_{0}=\frac{1}{2\pi f_{5}\cdot R_{123}}.$$

Using the method described above, values of the circuit parameters have been calculated from the plot given in Figure 6. The results are compared with those obtained using the commercial software EqCwin applied to the data from Figure 6 (Table 1).

The plot in Figure 7 gives similar results, except R_3_, that is, in this case, 150 Ω. Plots in Figures 6 and 7 do not have the fourth plateau for highest frequencies, so R_0_ could not be determined from such a curve.

## 4. Conclusions

Table 1 shows a very good agreement between the actual values of the electrical components forming the investigated physical model, the values obtained by the method described in this work and the values obtained using a commercial software product. In that way the method, hardware and software are fully confirmed. It should be emphasized that this method describes the system very well and clearly, but its big disadvantage is that it works with very low frequencies (on the order of μHz), that means a need for special equipment (like this described in the present work, or similar) and the experiments have a very long duration. The second problem may be resolved by starting the experiment from a frequency f_2_ (much higher than previously indicated), but in that case R_3_ must be determined by some other method (for example potentiostatic).

## Figures and Tables

**Table 1.** Parameters of the investigated equivalent circuit.

**Parameter**	**Actual value**	**Measured value**	**EqCwin value**
R_1_ [Ω]	39	41	45
C_1_ [F]	0.03	0.03	0.028
R_2_ [Ω]	90	93	93.4
C_2_ [F]	1.6	1.58	1.59
R_3_ [Ω]	1,000	992	1,003
