# Correction: Stević, Z. *et al*. Estimation of Parameters Obtained by Electrochemical Impedance Spectroscopy on Systems Containing High Capacities. *Sensors* 2009, *9*, 7365–7373

**DOI:** 10.3390/s90907397

**Published:** 2009-09-14

**Authors:** Zoran Stević, Mirjana Rajčić Vujasinović, Aleksandar Dekanski

**Affiliations:** 1 University of Belgrade, Technical Faculty in Bor, VJ 12, 19210 Bor, Serbia; E-Mail: mrajcic@tf.bor.ac.rs (M.R.V.); 2 University of Belgrade, Institute of Chemistry, Technology and Metallurgy, Njegoševa 12, 11001 Belgrade, Serbia; E-Mail: dekanski@ihtm.bg.ac.rs

In the original published version of this paper [1], the name and affiliation of the third coauthor was listed by mistake, and should be as indicated below:

Zoran Stević^1,^*, Mirjana Rajčić Vujasinović^1^ and Aleksandar Dekanski^2^

^1^ University of Belgrade, Technical Faculty in Bor, VJ 12, 19210 Bor, Serbia; E-Mail: mrajcic@tf.bor.ac.rs (M.R.V.)

^2^ University of Belgrade, Institute of Chemistry, Technology and Metallurgy, Njegoševa 12, 11001 Belgrade, Serbia; E-Mail: dekanski@ihtm.bg.ac.rs

## References

[1] Stević Z., Vujasinović M.R., Radunović M. (2009). Estimation of Parameters Obtained by Electrochemical Impedance Spectroscopy on Systems Containing High Capacities. Sensors.

